# Evaluating Billing Code Distributions in the Emergency Department Following the Implementation of the New Documentation Guidelines

**DOI:** 10.1016/j.acepjo.2024.100034

**Published:** 2025-01-31

**Authors:** Adriana Coleska, Melissa A. Meeker, Joseph W. Kopp, Phillip L. Rice, David S. Huckins, Karen L. Smith, Lauren M. Nentwich, Ali S. Raja, Joshua J. Baugh

**Affiliations:** 1 Department of Emergency Medicine, Massachusetts General Hospital, Harvard Medical School, Boston, Massachusetts, USA; 2 Department of Emergency Medicine, Brigham and Women’s Hospital, Harvard Medical School, Boston, Massachusetts, USA; 3 Department of Emergency Medicine, Salem Hospital, Boston, Massachusetts, USA; 4 Department of Emergency Medicine, Newton-Wellesley Hospital, Tufts University Medical School, Boston, Massachusetts, USA; 5 Coding Department, Massachusetts General Hospital, Boston, Massachusetts, USA

**Keywords:** documentation, billing, coding, emergency medicine

## Abstract

**Objectives:**

Changes to the Current Procedural Terminology (CPT) evaluation and management (E/M) documentation guidelines implemented on January 1, 2023, were primarily meant to address dissatisfaction with the prior system; however, it was not known how the changes might alter billing distributions. In this study, we compare the proportion of visits for each E/M code before and after the enactment of the changes across 5 emergency departments (EDs) to determine the effects on billing.

**Methods:**

This was a retrospective, observational analysis of all ED visits for patients over 18 years across 5 EDs from January 1 to March 31 in the years 2021, 2022, and 2023. In the primary analysis, we compared the distribution of visits for each of the studied CPT E/M codes in the 3 months before and after the enactment of the changes, utilizing a multivariate mixed-effect Poisson regression model. In our secondary analysis, we aimed to determine if the results differed when looking at academic and community sites separately.

**Results:**

Across all hospitals, visits coded as level 4 and level 5 comprised a significantly higher proportion of all visits in the postimplementation period (relative risk = 1.40 for level 4 and relative risk = 1.17 for level 5). The proportion of visits coded as levels 1, 2, and 3 significantly decreased in the postimplementation period, while those coded as critical care did not change. The same general trends were found in both academic and community settings separately, although with less statistical significance, particularly at the academic sites.

**Conclusion:**

In this observational analysis, we found that overall CPT E/M levels increased after the implementation of the new documentation guidelines, relieving apprehension that the documentation changes may lead to a decrease in reimbursement.

**The Bottom Line**

This retrospective observational analysis sought to determine whether the changes to the Current Procedural Terminology evaluation and management documentation guidelines implemented on January 1, 2023, brought forth changes to the distribution of Current Procedural Terminology evaluation and management levels in emergency medicine and, if so, how they changed. We found an increase in visits coded as level 4 and 5 (relative risk (RR) = 1.40 for level 4 and RR = 1.17 for level 5), with a concomitant decrease in visits coded as level 1, 2, and 3 (RR = 0.07, RR = 0.12, and RR = 0.25, respectively).

## 1 Introduction

### 1.1 Background

Changes to the Current Procedural Terminology (CPT) evaluation and management (E/M) documentation guidelines implemented January 1, 2023, represent the first big shift in documentation in emergency medicine (EM) since 1995. The impetus behind the changes was to decrease the administrative burden on clinicians and “note-bloat,” as well as to decrease the need for audits.^1^ The new documentation paradigm focuses on the physician’s thought process throughout clinical encounters, moving away from prior numerical standards that encouraged copy/paste and rote box-checking.^2^ The 2023 guidelines removed the mandatory elements of history, physical, and review of systems, instead encouraging physicians to only include what they feel is medically relevant. The selection of levels of services is now based on a variety of elements in the medical decision-making portion of the note.^3^

### 1.2 Importance

The documentation changes were primarily meant to address dissatisfaction with the prior system, as studies have shown that the documentation burden has contributed to burnout in EM.^4^ However, it was not known how the changes might alter billing and coding distributions, which have important implications for emergency department (ED) operations and patients alike. Literature from internal medicine suggests that recent documentation guideline changes in the outpatient setting have led to a shift toward higher-level codes, but the impact on EM has not yet been explored.^5^

### 1.3 Goals of This Investigation

In this study, we compare the proportion of visits for each E/M code before and after the enactment of the new guideline changes across 5 EDs within a large health care system to determine the effects on billing.

## 2 Methods

### 2.1 Study Design and Setting

This study was a retrospective observational analysis of data from 5 EDs in one large integrated health care system in the Northeastern United States, including 2 quaternary-care academic EDs and 3 community hospital EDs. The total combined adult visits per year for the represented academic centers is approximately 131,000. The total combined number of adult visits per year for the community sites is approximately 125,000. The ED CPT E/M codes studied were 99281, 99282, 99283, 99284, 99285, and 99291, also known as levels 1, 2, 3, 4, 5, and critical care, respectively. Both academic centers use a combination of attending physician, resident, and advanced practice provider (APP) documentation to assign a CPT E/M code for each visit. Two of the 3 community sites do not have residents and use a combination of attending physician and APP documentation to assign billing levels; the third community site has attendings, residents, and APPs. Anecdotally, <1% of all academic charts reviewed were APP-only visits vs <10% at the community sites. E/M levels are assigned by 3 different billing and coding companies across the 5 sites. This study was deemed to not meet the criteria for human subject research according to institutional guidelines.

Prior to and throughout the January rollout, the EDs implemented a variety of educational initiatives to help their attending physicians, residents, and APPs become more familiar with the new guidelines. Each site provided a documentation session led by faculty and/or their coder group introducing the new guidelines. Physicians and APPs were also provided with videos outlining the changes, a “Common Questions” tip sheet, and electronic medical record dot phrases to help implement the documentation changes. Finally, 3 of the 5 sites held small group sessions run by faculty to allow for open discussion and direct feedback on charts.

### 2.2 Study Population and Data Collection

All ED visits for patients over the age of 18 were included from January 1 to March 31 in the years 2021, 2022, and 2023. Data were extracted from coder databases and included the number of visits assigned to each ED CPT E/M code for the above-mentioned months at each of the 5 sites. The data were extracted in aggregate for each month; no individual visit data were collected.

### 2.3 Statistical Analysis

The primary outcome measure was the count of visits to the ED for the relevant CPT E/M codes. The explanatory variable of interest was the time period pre- and postimplementation of the new documentation guidelines. Specifically, we compared the distribution of visits with the studied CPT E/M codes in January-March of 2023 with the distribution of visits with the same codes in January-March of 2021 and 2022 across all 5 EDs. Our model data included an individual month-site value for each E/M level in each year. With 6 E/M codes, 5 sites, 3 months, and 3 years, this led to a dataset with 270 values. First, we utilized a chi-square analysis to assess for any significant differences in overall coding distribution before and after the documentation changes. Second, we used a multivariate mixed-effect Poisson regression model with an offset to test for significant differences in the distribution of visits for each CPT E/M code between the pre-and posttime periods. We used a multivariate model to account for the joint distribution between the outcome of each E/M code, and the offset was incorporated to control for changes in total visit volume over time. Of note, we included code 99291 – the CPT E/M code for critical care – as a control, given that the new guidelines should not have had any impact on critical care documentation or billing.

For our secondary analysis, we performed the same regression analyses as above, stratified by site type: academic and community.

## 3 Results

### 3.1 Distribution of Visits for the Studied CPT E/M Codes

**Figure 1:**
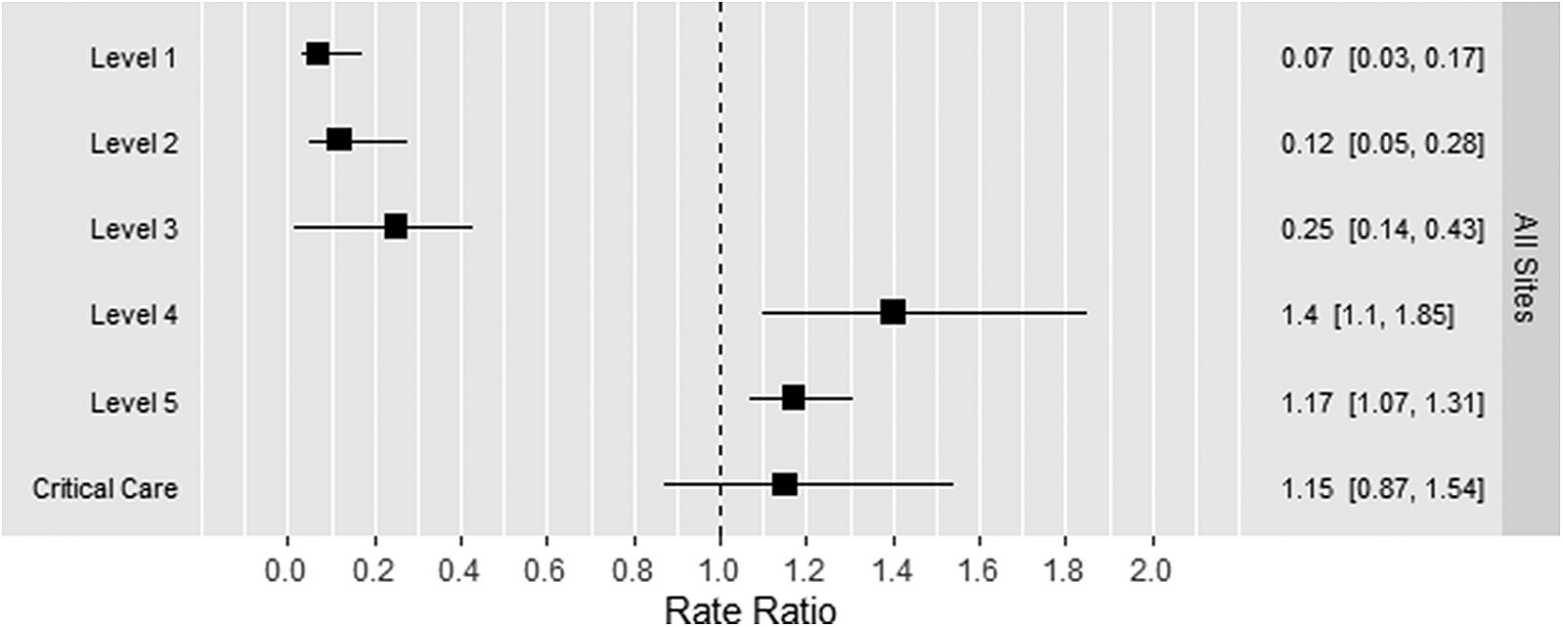
Multivariate regression analysis to assess for changes in evaluation and management levels from January to March 2023 compared with the same months in 2021 and 2022 across all 5 study sites. An estimate of 1 represents no difference in the outcome before and after the implementation. An estimate of <1 represents a decrease, and an estimate of >1 represents an increase in the outcome before and after the implementation. Box and whisker plots that do not cross the dotted line at a rate ratio of 1 signify statistically significant results.

**Figure 2:**
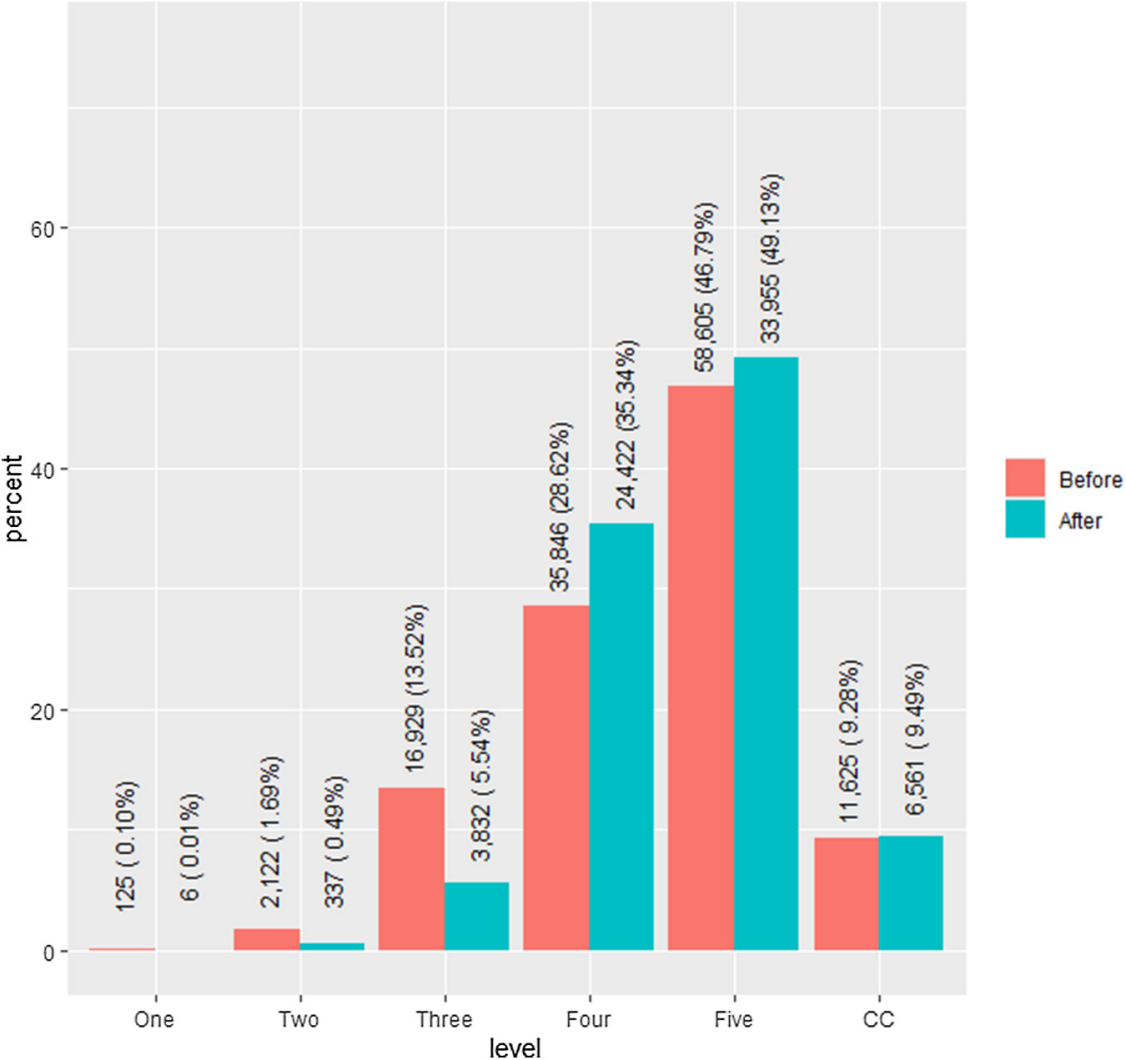
Raw numbers and percentages of visits across studied levels before and after the documentation changes across the full study sample. CC, critical care.

Across all hospitals, the chi-square analysis demonstrated that the distribution of coding levels was significantly different in the postimplementation period (*P* < .001). From the multivariate analysis, there was an estimated 1.40 and 1.17 times higher rate of visits coded as level 4 and level 5, respectively, in the postimplementation period compared with the preimplementation period (95% CI [1.10, 1.85] and 95% CI [1.07, 1.31], respectively) (Fig 1). In raw percentages, level 4 and level 5 visits increased by roughly 7 and 1.5 percentage points, respectively (Fig 2). In contrast, there was an estimated 0.07, 0.12, and 0.25 times lower rate of visits coded as level 1, level 2, and level 3, respectively, in the postimplementation period compared with the preimplementation period (95% CI [0.03, 0.17], 95% CI [0.05, 0.28], and 95% CI [0.14, 1.31], respectively) (Fig 1). In raw percentages, levels 1, 2, and 3 decreased by roughly 0.1, 1, and 8 percentage points, respectively. Finally, there was no statistically significant change in the rate of visits coded as critical care in the post- vs preimplementation periods.

### 3.2 Differences Between Academic and Community Sites

**Figure 3:**
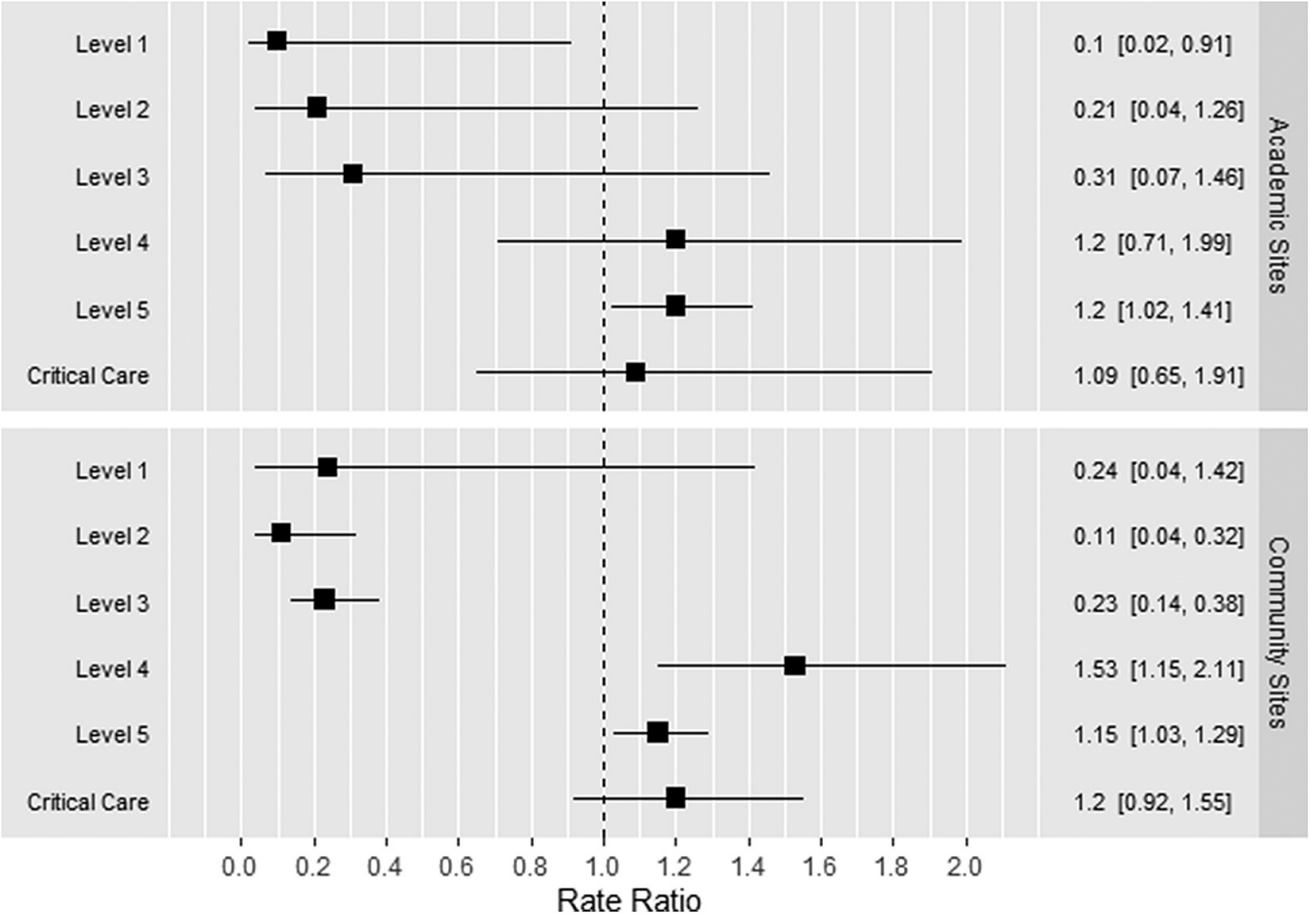
Multivariate regression analysis to assess for changes in evaluation and management levels from January to March 2023 compared with the same months in 2021 and 2022, separated by type of site: academic vs community. An estimate of 1 represents no difference in the outcome before and after the implementation. An estimate of <1 represents a decrease, and an estimate of >1 represents an increase in the outcome before and after the implementation. Box and whisker plots that do not cross the dotted line at a rate ratio of 1 signify statistically significant results.

As seen in Figure 3, the same general trends were found in both academic and community settings separately, although with less statistical significance, particularly at the academic sites. For community sites, the proportion of visits coded as levels 4 and 5 comprised a statistically significantly higher proportion of visits in the postimplementation period compared with the preimplementation period, and the proportion of visits coded as levels 2 and 3 had a statistically significant decrease in the postimplementation period compared with the preimplementation period. Community sites did not see a statistically significant change in visits coded as level 1. At the academic sites, an increase in visits coded as level 5 and a decrease in visits coded as level 1 was the only statistically significant differences in the postimplementation period compared with the preimplementation period, while changes in levels 2 to 4 were directionally the same as the overall trends but not statistically significant. Visits coded as critical care did not change significantly in either analysis.

## 4 Limitations

Our study had several limitations. First, our conclusions are based on 3 months of data under the new coding guidelines. Despite the short timeframe, we believe that publishing this early trend will help physician groups across the nation understand the potential implications of the new guidelines. Second, our data are drawn from a single health care system, potentially limiting generalizability. However, it is notable that the study included academic and community EDs of varying sizes and that similar results were noted across both types of sites. Furthermore, 3 different billing and coding companies were used across the 5 EDs to determine the code level for each visit, suggesting that our results could not be explained by the practices of a single coding entity. Finally, the data were collected from the first 3 months following the documentation change. It is possible that as clinicians become more comfortable with the documentation changes, the visit levels may shift even more drastically. Further studies will be needed that explore a longer timeline.

## 5 Discussion

In this analysis of ED billing code distributions before and after the implementation of the new documentation guidelines, we found an increase in visits coded as levels 4 and 5, with a concomitant decrease in visits coded as levels 1, 2, and 3. No statistically significant changes were noted in the proportion of visits coded as critical care visits. Our secondary analysis may suggest that these changes were more pronounced at community sites vs academic settings; however, given the small sample size, this subanalysis should be interpreted with caution. Furthermore, given the higher level of care offered at academic sites, it is expected that academic centers would have a patient population that is skewed toward higher complexity of care, leading to higher levels of visits.

The changes in level distribution observed may be explained by the addition of coding elements that better account for the overall process of treating a patient, such as data gathered from multiple sources, actions considered, and thought processes described. For example, visits for low acuity musculoskeletal injuries that were previously coded as a level 1 or 2 may now qualify for a level 3 by including details regarding why imaging was not ordered, why a narcotic prescription was considered but not given, and so on. Similarly, accounting for personally interpreted imaging and frequently administered medications like ondansetron, parental narcotics, and intravenous contrast, now considered actions that increase the risk of complications and/or morbidity or mortality of patient management, can elevate visits that were previously coded as level 3 or 4 a level or 2 higher. As the 2023 CPT E/M documentation guidelines did not make any changes to critical care documentation, our findings that the proportion of visits coded as critical care did not change was reassuring that our results were driven by the changes from the new documentation rules rather than changes in patient acuity.

Changes in documentation guidelines can lead to apprehension that there will be an unwanted change in billing and a concomitant decrease in overall reimbursement. This analysis indicates that the January 1, 2023 documentation guidelines have led to an opportunity for higher reimbursement rates in EM. Interestingly, this trend matches what has been seen in outpatient practices in internal medicine thus far after analogous changes were made to outpatient documentation guidelines in 2021.^4^ While these trends will be welcome news to health care practices in EM and outpatient fields, it is not clear how changes in coding distribution might ultimately affect how public and private insurers set reimbursements for various E/M codes.

It is worth noting the effort that sites in our study put toward educating the clinical staff; this likely contributed to the favorable trend as the new guidelines represented a drastic shift from the prior documentation criteria.^6^ Settings with less educational efforts might find different results. Prior research has shown that inaccurate billing and coding can have important repercussions for EDs, including lost revenue and legal consequences, making education about the new guidelines very important.^7^

In this observational analysis, we found that overall CPT E/M levels increased after implementation of the new documentation guidelines, with increases in visits coded as level 4 and 5 and concomitant decreases in those coded as level 1, 2, and 3. With no significant change observed in critical care visits, our results suggest that the shifts in coding levels were due to the new guidelines rather than a change in true patient acuity. Given that higher coding levels result in higher levels of billing, EDs may see an increase in revenue after the implementation of the new guidelines. Further studies should assess if these findings are generalizable and durable and if there is a real difference in percentages of CPT E/M levels between academic and community sites. Furthermore, additional studies should investigate whether the qualitative goals of the new CPT E/M documentation guidelines were realized.

## Author Contributions

A.C. and J.J.B. developed the study design. A.C. gathered the data and drafted the manuscript. A.C., J.J.B., and M.A.M. discussed the plan for data analysis. M.A.M. analyzed the data. J.K., P.L.R., D.S.H., K.L.S., L.M.N., A.S.R., and J.J.B. provided critical revisions of the manuscript for important intellectual content.

## Funding and Support

By *JACEP Open* policy, all authors are required to disclose any and all commercial, financial, and other relationships in any way related to the subject of this article as per ICMJE conflict of interest guidelines (see www.icmje.org). The authors have stated that no such relationships exist.

## Conflict of Interest

A.C., J.W.K., P.L.R., D.S.H., K.L.S., L.M.N., A.S.R., and J.J.B. report no conflicts of interest.
